# A semi‐active human digital twin model for detecting severity of carotid stenoses from head vibration—A coupled computational mechanics and computer vision method

**DOI:** 10.1002/cnm.3180

**Published:** 2019-02-20

**Authors:** Neeraj Kavan Chakshu, Jason Carson, Igor Sazonov, Perumal Nithiarasu

**Affiliations:** ^1^ Biomedical Engineering Group, Zienkiewicz Centre for Computational Engineering, College of Engineering Swansea University Swansea SA2 8PP UK

**Keywords:** biomechanical vibrations, blood flow, carotid stenoses, computer vision, digital twin, face video, systemic circulation

## Abstract

In this work, we propose a methodology to detect the severity of carotid stenosis from a video of a human face with the help of a coupled blood flow and head vibration model. This semi‐active digital twin model is an attempt to link noninvasive video of a patient face to the percentage of carotid occlusion. The pulsatile nature of blood flow through the carotid arteries induces a subtle head vibration. This vibration is a potential indicator of carotid stenosis severity, and it is exploited in the present study. A head vibration model has been proposed in the present work that is linked to the forces generated by blood flow with or without occlusion. The model is used to generate a large number of virtual head vibration data for different degrees of occlusion. In order to determine the in vivo head vibration, a computer vision algorithm is adopted to use human face videos. The in vivo vibrations are compared against the virtual vibration data generated from the coupled computational blood flow/vibration model. A comparison of the in vivo vibration is made against the virtual data to find the best fit between in vivo and virtual data. The preliminary results on healthy subjects and a patient clearly indicate that the model is accurate and it possesses the potential for detecting approximate severity of carotid artery stenoses.

## 1 INTRODUCTION

The digital twin concept is becoming a common theme in traditional engineering disciplines, and such a concept is yet to be completely realised in cardiovascular engineering. The digital twin concept can be broadly described by three characterisations: active, where a digital replica (digital twin) of a physical system (physical twin) is continuously updated by information and data collected from the physical twin; semi‐active, where time‐varying data are collected, but rather than performing a continuous update, the information is analysed after the data are collected; and passive, where the digital twin utilises measurements from a physical twin which are not continuously updated, which may include some modelling assumptions. It is also possible to have a mix of active and passive digital twin models, where only specific sections or parameters of the digital twin are continuously updated via data collected from a physical twin, while other components of the model either use assumptions, or utilise measurements from a physical twin but are not being continuously updated. The active and passive digital twin concepts of the systemic circulation are currently being considered by researchers.^1^, ^2^ While the passive digital twin concept has been realised through off‐line calculations in cardiovascular flow modelling, the active concept is fairly new. These passive concepts include off‐line fractional flow reserve (FFR) calculations^3^, ^4^, ^5^ and a large number of subject‐specific blood flow calculations through aneurysms and stenoses. The active digital twin has all the ingredients to be the basis for future noninvasive diagnostic methods of cardiovascular problems, as we are increasingly making active and continuous online measurements of subject‐specific cardiac signals. An active FFR calculation would require producing an FFR value instantaneously while a scan of the a coronary artery is being carried out. With the fast computational methods and emerging machine learning algorithms, we believe that such an active digital twin model is now plausible. In the present work, we attempt a semi‐active digital twin model for noninvasively detecting a carotid artery stenosis. In this method, a time‐dependent face video of a subject is used to calculate the head vibration before comparing the in vivo value to computationally generated data, to approximately determine the severity of a carotid artery stenosis.

The carotid arteries are the main vessels that carry blood to the head. Due to ageing, hypertension, life style choices, and injuries to the blood vessel wall, plaques build over time in the carotid artery wall layers. This is called atherosclerosis, and it causes a progressive narrowing of carotid artery, known as carotid stenosis. As the plaque builds up, the inward growth of mass narrows the internal lumen diameter. Such a narrowing of the carotid artery can lead to reduced blood supply, and hence oxygen supply, to the brain. This reduction in oxygen supply may cause the death of brain tissue, leading to ischemic strokes or transient ischemic attacks (TIA). Annually, 16 million people suffer from stroke around the globe,^6^ making it the third highest cause of death in the world after cancer and coronary heart disease (CHD). In the United Kingdom alone, 85% of the 100 000 cases reported were ischaemic in nature.^7^ In majority of the cases assessing the severity of carotid narrowing after a TIA is still a major challenge. The current assessment procedure of carotid duplex ultrasound often is delayed due to waiting time and other issues. Thus, development of other easier and noninvasive methods will add value to existing screening technologies. In other developing countries, availability of medical devices is very limited and a procedure like the one proposed can provide low cost screening of suspected TIA patients. Furthermore, there are other perceived barriers to the use of ultrasound in developing countries, which include lack of training or training opportunities; unable to afford the cost of obtaining, maintaining, or updating the equipment; and lack of reliable electricity supply.^8^ Furthermore, ultrasound is dependant on the Doppler that can influence the peak systolic velocity value.^9^ Thus, development of a more consistent and reliable method would be useful to estimate stenosis severity. Conventionally carotid stenosis is clinically detected using either a doppler ultrasound of the neck, magnetic resonance angiography (MRA), CT angiography (CTA) of the neck, or a cerebral angiography. In the present work, a nonconventional method is proposed.

The forces generated as a result of pulsatile blood flow in major arteries lead to low amplitude vibrations of human body parts. These vibrations are potentially a vital sign for assessing the health of arteries. Many noninvasive methods to detect and analyse such vibrations have been developed, such as Seismocardiography, kinetocardiography, and ballistocardiography. Out of these, ballistocardiography (BCG) was once a topic researched upon extensively but has been falling out of favour over time due to a lack of sophisticated and accurate measuring equipment and techniques. A detailed analysis of the development of BCG and the reasons for why it was discarded have been well documented by Giovangrandi et al.^10^ Proposed in the 19th century, BCG was given importance in 1940s to 1980s. Originally, setups such as sensitive vibration beds were developed to capture these vibrations. It has again gained momentum in 2010 after accurate and more sensitive sensors and vibration measuring devices have emerged. A type of BCG that records head movements or vibrations in the head due to blood flow, also known as head‐BCG, was analysed by He et al.^11^

Blood flow, primarily in the carotid arteries, causes subtle head motions. Occlusion in these arteries can cause changes in this head motion due to a substantial change in amplitude of pressure waves in comparison to nonoccluded arteries. A procedure capable of detecting very subtle movements can be used for accurately capturing these motions, which cannot be sensed by our naked eye is essential to make progress. One such powerful tool is computer vision, which can analyse extremely small motions in a video. This has been made possible by advancements in camera technology and the raw power of processing. In Balakrishnan et al,^12^ computer vision was used to capture this subtle head motion to determine heart rate and variability. In older computer vision algorithms, the pulse was detected using colour change in the skin. One of the future research possibilities mentioned in the past was to detect blockages in arteries, which provided a motivation to investigate the possibility of using cameras to detect the severity of carotid stenoses. This type of predictive methodology could potentially prevent strokes as widely accessible devices such as smart phones could be used for screening.

**Figure 1:**
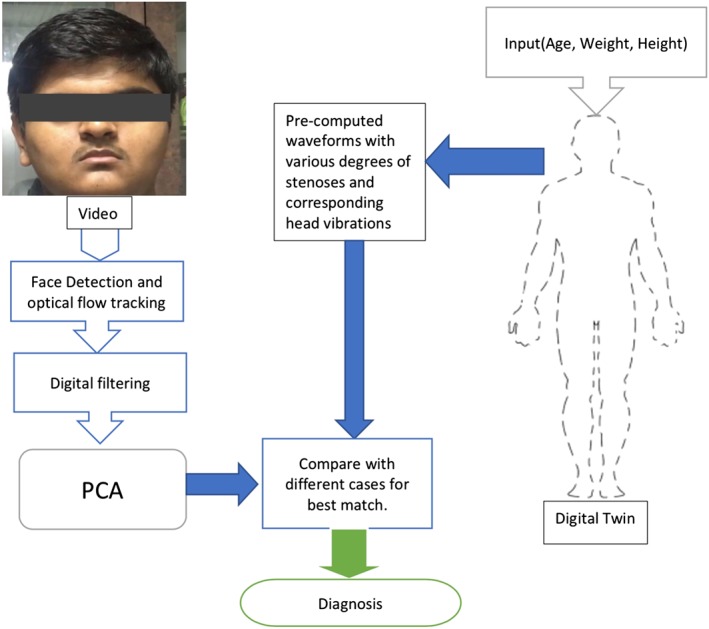
Diagnosis of carotid stenosis by comparing results of computer vision and biomechanical model (digital twin)

In the present work, we are attempting to mechanically model head vibrations and then use the model to predict vibrations corresponding to carotid stenoses. The proposed methodology has the following steps: (a) Generate synthetic head vibration data (computational) for different degrees of carotid artery occlusions by combining a systemic circulation blood flow model and a head vibration model; (b) Estimate the vibration of a human head as a result of blood flow by analysing the face video via a principal component analysis; and (c) Compare and match the measured vibration against the synthetic data to come to a conclusion on the severity of carotid artery occlusion. These steps are schematically presented in Figure 1.This paper is organised into following sections. In the section that follows the introduction, analysis of face video is discussed. In Section 3, the methodology used to create the synthetic data is discussed in detail. This includes the blood flow model, vibration model, and the interface between these two models. In Sections 4 and 5, respectively, some preliminary results and limitations of the present work are discussed, and finally Section 6 provides some important conclusions and potential future research directions.

## 2 DETECTION OF HEAD OSCILLATIONS

An algorithm proposed by Balakrishnan et al^12^ that uses computer vision to detect small motions in the head has been implemented in this work. A few modifications have been incorporated to enhance and simplify small motion analysis and help detect stenoses, such as changing the region of interest from part of the face to only the forehead region. The proposed methodology analyses between 30  and 40 seconds of captured video. The analysis is not carried out in real time and thus we refer to the method as “semi‐active digital twin.” A smart phone–based camera was used to improve accessibility, which could eventually provide a low‐cost and noninvasive technique for detection of carotid occlusion. The videos were captured on a Motorola G (second generation) for android devices, which comes with an 8MP camera, and an iPhone 6s with 12MP camera, for iOS devices. Due to its built‐in features such as higher resolution, the iPhone 6s provides a better solution. In this work, samples were captured using both cameras.

### 2.1 Assumptions and guidelines

A basic assumption has been made that the subject remains still. Most of the involuntary actions such as respiration and blinking of eyes are removed digitally when the signal is passed through a bandpass filter. For an accurate detection of motion during the video recording, the following set of guidelines are followed:

- The subject has been rested sufficiently before screening to ensure a relatively stable heart rate and respiration rate.
- The video is shot in an environment with no direct lighting over the subject's face which ensures reliable feature tracking.
- Neither the subject nor the camera is subjected to any small vibrations. For example, a camera placed on a table with a desktop computer can produce erroneous results.

### 2.2 Facial recognition and region of interest

**Figure 2:**
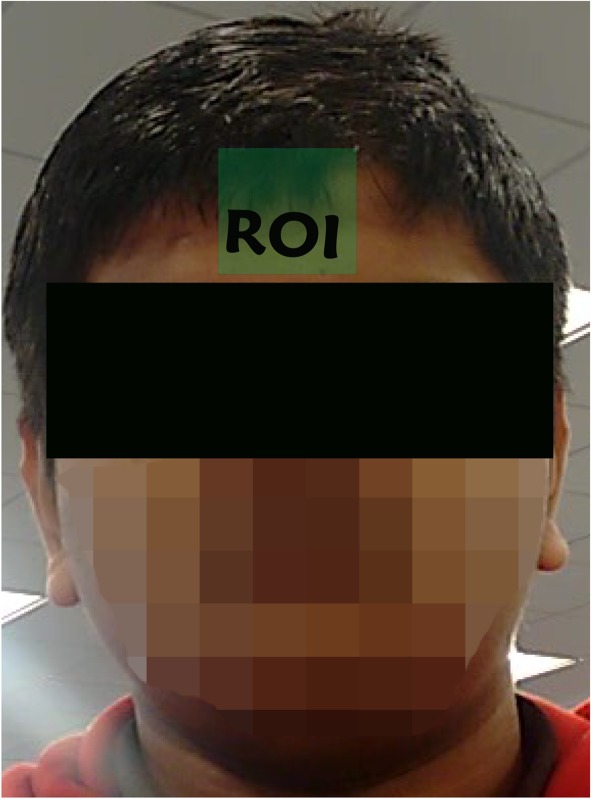
Region of interest

In order to detect the region of the video covered by the face, the Viola‐Jones face detector was used.^13^, ^14^ This detector provides an object detection framework which allows competitive object detection rates in real time. A small region, usually the central forehead or area below the eyes, is taken as the region of interest (although regions from other parts of the face may also work successfully). Unlike in Balakrishna et al,^12^ in which 50% to 60% of the face width and 60% to 70% of the face length was selected, only a small rectangular (forehead) region has been used in the present work (see Figure 2). This helps in maintaining a consistent average distance between the pivoting point at the base of the neck and the monitoring points, simplifies the conversion of angular displacement to linear displacement, as explained in Section 3.

### 2.3 Feature points tracking and filtering

**Figure 3:**
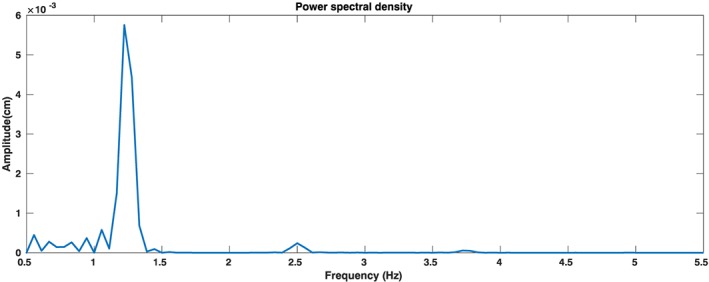
Energy at different frequencies for the results of blood‐flow‐driven‐head‐neck vibration predicted by the proposed mechanical model

OpenCV Lukas‐Kanade optical flow^13^ was used to track a number of points (N‐points) within the region of interest. Since two components of motion, vertical and horizontal, can be detected during tracking, selection of a component for analysis and detection becomes necessary. It was noted by both Balakrishna et al^12^ and He et al^11^ that the horizontal motion was mainly due to dynamic equilibrium swaying. Thus, the horizontal component has been ignored in the present work. The vertical component of the signal is then passed through a Butterworth bandpass digital filter. The frequency band for this filter was chosen to be 0.75 to 2 Hz. This frequency range was derived from a spectral analysis, using fast Fourier transforms, of predicted head‐neck vibration from the mechanical model proposed in this work. Figure 3 shows energy at different frequencies from the results of blood‐flow‐driven‐head‐neck vibration predicted by the proposed mechanical model.The frequencies being observed are above 0.5 Hz to avoid interference from very low frequency vibrations caused by different neurological activities and dynamic equilibrium swaying. Filtering of vertical signal (from the video) gave an output very close to that of the predicted results (from the mechanical model). This frequency range is also the same as a heart beat range, where 0.75 to 2 Hz can be related to 45 to 120 beats per minute.

### 2.4 Principal component analysis

**Figure 4:**
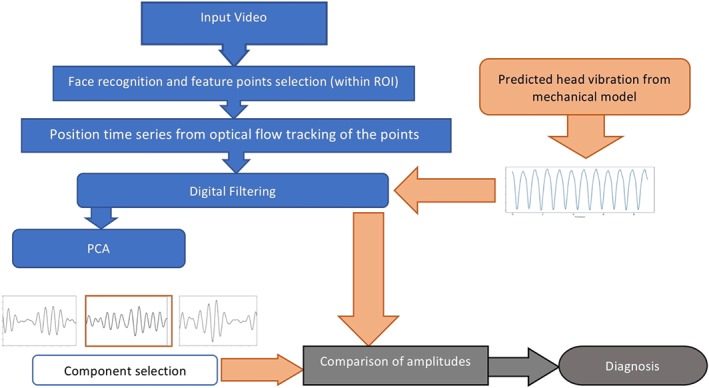
Workflow used in the present work to detect carotid stenosis and it's severity

In the present work, we are interested in head vibrations that are driven by haemodynamics. However, as head motion is caused by several factors, which includes blood flow, respiration, facial expressions, and neurological processes, it is necessary to separate the components of this mixed head motion into submotions in order to isolate blood flow driven vibration. This is carried out by performing a principal component analysis (PCA) using a similar technique to that of Balakrishna et al.^12^ Later works^15^, ^16^, ^17^, ^18^ have implemented other methods, such as the discrete cosine transform, in order to isolate different types of motion. However, they generally gave results close to PCA, and thus, PCA is chosen in the present work due to its simplicity(see Figure 4).

PCA is described by the following algorithm: let *y*_*fn*_ be the vertical displacement of the *n*th point at the *f*th frame: *n*  =  {1,…,*N*} and *f*  =  {1,…,*F*} where *N* is the number of points accounted and *F* is the total number of frames in the recording. We define the mean as follows:

$$\mu_{n}=\frac{1}{F}\sum_{f=1}^{F}y_{fn},$$

and the matrix ***Y*** of centred displacements with the entries as follows:

$$Y_{fn}=y_{fn}-\mu_{n}.$$

We define the covariance matrix as follows:

$$C=\frac{1}{F-1}YY^{T}.$$

The PCA finds the principal axes of variation of the position as the eigenvectors of the covariance matrix from

CU=ΛU,

where **Λ**=diag{*λ*_1_,…,*λ*_*N*_} is a diagonal matrix of the eigenvalues and $U=\left[u_{1},\text{⋯},u_{N}\right]$ is a matrix with each column of it being an eigenvector, ***u***_*n*_, corresponding to the eigenvalue *λ*_*n*_. In our work, only the eigenvectors are of interest and not eigenvalues.

The final required signal, in the form of head displacement, can be written as follows:

$$S_{i}(t)=y_{fn}u_{i},$$

where *t*  =  *f*Δ*t* with Δ*t* being the time‐step between two neighbouring frames and *i* in the above equation represents the eigenvector of interest.

The eigenvector (principal component) of interest is selected by analysing two properties of time series signals calculated from different eigenvectors. A signal having a frequency corresponding to the heart rate of the subject with good periodicity (reflecting a healthy heart rate variability) is chosen as the component of interest for the healthy condition, without the presence of a carotid stenosis.

However, since the majority of stenosis cases occur in older subjects and thus it is highly likely to be coupled with other cardiovascular diseases, an ambiguity may arise when choosing the eigenvector of interest for these subjects; thus, a different technique must be performed. In these subjects, the head‐neck vibration is not only affected by heart rate but is also significantly affected by factors such as cardiac output, heart rate variability, and the occurrence of multiple stenosis or aneurysms. The heart rate variability, unlike in healthy conditions, leads to a nonperiodic signal. For these cases, component selection requires more sophisticated and trial‐tested techniques such as machine learning, where multiple input parameters can be used to select the required component. However, such techniques require substantial amounts of data that can only be collected through an extensive retrospective study; hence, in this work, the selection of components for all subjects, including the stenosis patient, was performed by analysing only the frequency and heart rate variability of the subject. Although the same technique was implemented for the selection of eigenvectors for both healthy subjects and the stenosed subject, the stenosis patient was on medication for CVD and had been treated for irregular heartbeats, thus making the signal relatively periodic.

## 3 MECHANICAL MODELLING OF HEAD OSCILLATIONS

A mechanical model is required to produce a database of virtual patients. The database surrogates the data required for developing an automated detection system, which otherwise would require a significant number of patients suffering from different forms of carotid stenoses. It also serves as a reference for choosing the number of filtration levels and types of digital filters, along with refinement of the virtual patient parameters.

**Figure 5:**
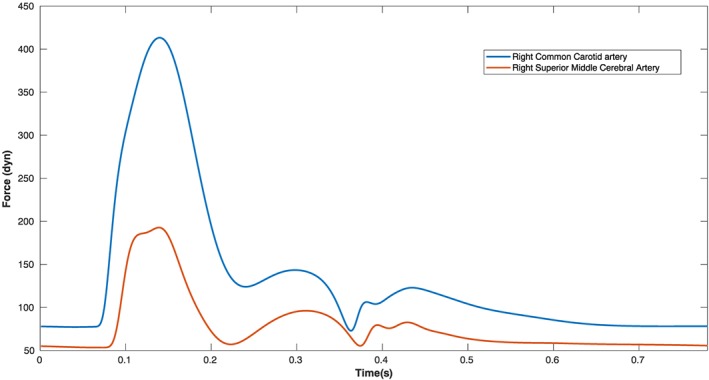
Force exerted by blood flow in a single finite element of two major inter‐cranial arteries with respect to time

The arterial network present in the head‐neck system is extremely complicated, and analysing each of the arteries for their contribution to the head‐neck oscillations is a daunting task, particularly as there are anatomical variations of the cerebral arteries. In order to reduce the complexity, forces imparted by various inter‐cranial arteries were analysed using the one‐dimensional blood flow model, and it was observed that blood flow in the carotid arteries (Figure 5) have the most significant influence on the subtle head oscillations. The other smaller inter‐cranial arteries had a very small influence when considered independently . However, when considered collectively, they have a small but significant contribution to the head‐neck vibration. Based on these observations, instead of accounting for the effect of each of these smaller arteries separately, we have mimicked their collective effect by scaling the force in the distal end of the internal carotid arteries. Since the majority of stenoses in this region develop in the carotid arteries, we chose to reduce the complexity of modelling by limiting the analysis to the carotid arteries. In the future, when more powerful graphic processing units and robust computer vision algorithms are available, the complexity of this model can be increased by including the individual effect of each of the other smaller arteries, especially those in the circle of Willis.

### 3.1 One‐dimensional haemodynamic model

The haemodynamic model adopted in this work is a modified version of the model proposed by Mynard and Smolich.^19^ In this work, only the systemic arteries are of interest, and so the systemic veins and pulmonary systems have been neglected. The model considers 123 of the major vessels in the systemic arteries as one‐dimensional vessel segments. The inlet of the aorta is connected to a two chamber zero‐dimensional heart model, while the outlet of peripheral vessels connects to a three‐element Windkessel model, which accounts for the microcirculation. A large number of alternative one‐dimensional models can be found in previous studies.^19^, ^20^, ^21^, ^22^, ^23^, ^24^, ^25^, ^26^, ^27^, ^28^, ^29^, ^30^, ^31^

#### 3.1.1 One‐dimensional vascular modelling, heart, and connectivity between vessels

Blood flow in the one‐dimensional vessel is governed by the nonlinear set of equations (Equations 1 and 2). An assumption of a flat velocity profile is used for the convective acceleration term, and a profile with a small boundary layer is chosen for the viscous friction term. A viscoelastic constitutive law is chosen for the walls, which consists of a power law model for the elastic term and a Voigt model for the viscous wall term (Equation 3). The wave speed from Equation 3, c_0_, is used to find the time required for the pulse to reach the carotid arteries. The majority of vascular beds in this model are treated using three‐element Windkessel models, which are constructed using (1) lumped compliances on the arterial side, (2) characteristic impedances coupling any number of connecting one‐dimensional arteries to the lumped parameter vascular bed, and (3) a constant vascular bed resistance to represent the downstream resistance of the microcirculation. These vascular bed models have been incorporated in all vascular beds except for the liver and myocardium. For a detailed discussion of the vascular bed modelling of liver and myocardium, see Mynard et al.^19^

**Figure 6:**
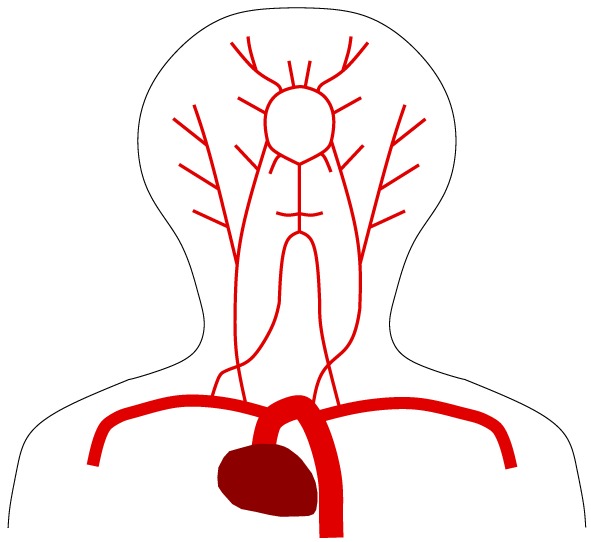
Cerebral arteries used in the one dimensional model

A zero‐dimensional (lumped model) of the heart is used in this model for the inlet boundary condition. Lagrange multipliers have been used to connect one‐dimensional vessels and are used to conserve mass and total pressure at vessel junctions. Conservation of mass and conservation of static pressure are used to connect the one‐dimensional and zero‐dimensional model. The system of equations are solved using the methodology in Carson and Van Loon,^29^ which is an implicit subdomain collocation scheme, and are

(1) $$\frac{\partial A}{\partial t}+\frac{\partial Q}{\partial x}=0,$$

(2) $$\frac{\partial Q}{\partial t}+\frac{\partial \left(Q^{2}/A\right)}{\partial x}+\frac{A}{\rho }\frac{\partial P}{\partial x}=-2\pi \gamma \nu \frac{Q}{A},$$

(3) $$P-P_{0}-P_{ext}=\frac{2\rho c_{0}^{2}}{b}\left[{\left(\frac{A}{A_{0}}\right)}^{b/2}-1\right]+\frac{\Gamma }{A_{0}\sqrt{A_{0}}}\frac{\partial A}{\partial t},$$

where Q is the volumetric flow rate, P is the hydrostatic pressure, P_ext_ is the external pressure, A is the lumen cross‐sectional area, t is the time, x is the axial coordinate, ρ is the density of blood, ν is the kinematic viscosity of the blood, γ is the viscosity parameter, Γ is the viscoelastic parameter and c is the intrinsic wave speed. Subscript 0 represents the diastolic condition. The length and diameter of common carotid arteries were modified according to the equations provided by Passera et al^32^ to approximate the vessel network that is specific to the subject. A part of the flow network used in the present study is shown in Figure 6.

### 3.2 Determination of axial force on the wall

Only the axial or unbalanced components of fluid force is used for determining the force applied on the walls. The radial forces are imparted on the arteries uniformly along the radial direction and thus cancel out any vibration resulting from these radial forces (in the calculation of the moment of force). In order to estimate the axial force on the wall, it is important to first discuss what is and what is not included in the model (or patient data), due to various necessary assumptions. There are significant variations in the range of parameters between individuals. This includes cardiac output (stroke volume), systolic and diastolic pressures, length and orientation of arterial segments, vessel lumen diameters, vessel wall parameters such as wall thickness, and the elastic modulus. An assumption in the haemodynamic model is that the vessel wall only moves in the radial direction.

It is necessary to simplify the estimation of axial force in such a way that the method is generalised. In order to attempt this, it is assumed that the axial force on the occluded/healthy vessel wall will be proportional to the net force of the fluid in the axial direction. The force of the fluid can be derived from considerations on a control volume.

**Figure 7:**
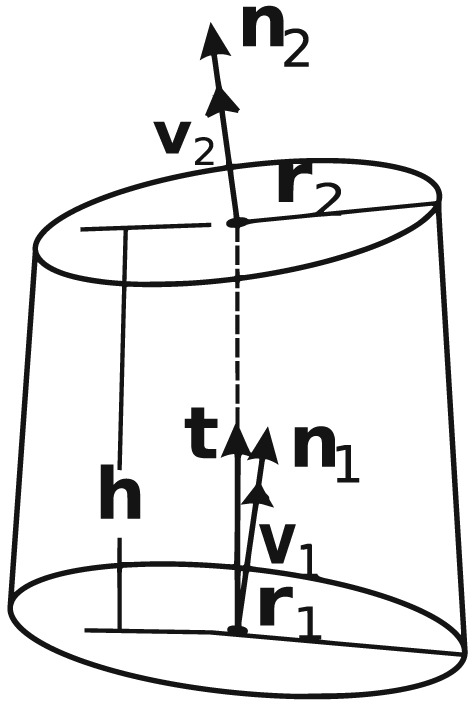
Element of the vessel of length h. Here, **t** is the unit vector acting along the element axis, **n**_1_ and **n**_2_ are the normals to the element inlet and outlet, respectively

Consider an element of a vessel segment shown in Figure 7. Let the element length be *h*, inlet radius is *R*_1_ and outlet radius be *R*_2_. Correspondingly, its inlet and outlet areas are $A_{1,2}=\pi R_{1,2}^{2}$. Let **t** be a unit vector acting along the element axis, **n**_1_ and **n**_2_ be normal unit vectors to the element inlet and outlet, respectively. If a segment is curved or it is the first or last in the segment then direction of inlet and outlet may be different and do not coincide with the direction of the element axis. We assume that angle between **n**_1_ and **n**_2_ is small enough and we can consider an element as truncated cone (if the segment is tapering) or a part of a cylinder. Let *v*_1_ and *v*_2_ be velocities averaged over inlet and outlet, respectively. The axial forces encountered by the wall are as follows:

1. Friction force **F**_*f*_ acting in direction **t**;
2. Force **F**_*d*_ caused by deflection of the flow in the element in the case **n**_1_ ≠ **n**_2_;
3. Force **F**_*t*_ caused by vessel tapering.

The friction force can be calculated as follows:

(4) $$F_{f}=F_{f}t,\;F_{f}=\tau A_{w},$$

where *τ* is the wall shear stress and *A*_*w*_ is the area of the element wall. The wall shear stress is given as follows:

(5) $$\tau =\gamma \mu \frac{v}{R}.$$

Here, *γ* is the friction coefficient, *μ* is dynamic viscosity, *v* is cross‐section average velocity and *R* is the cross‐section radius. The friction force per unit of length along the axis is *τ* times cross‐sectional perimeter 2*πR*:

(6) $$\frac{dF_{f}}{dx}=\gamma \mu \frac{v}{R}\times 2\pi R=2\pi \gamma \mu v.$$

Integrating (6) by the trapezoidal rule, we have

(7) $$F_{f}=\pi \gamma \mu \left(v_{1}+v_{2}\right)h.$$

The force acting on a curved pipe with the steady state flow is

(8) $$F_{d}=\left(v_{2}-v_{1}\right)\dot{m},$$

where $\dot{m}=\rho \bar{Q}$ is the mass flow rate and $\bar{Q}=\frac{1}{2}\left(A_{1}v_{1}+A_{2}v_{2}\right)$ is the average volumetric flow rate in the element. The normal component of this force can be approximated by

(9) $$F_{d}=\left(n_{2}-n_{1}\right)\bar{v}\;\dot{m}=\left(n_{2}-n_{1}\right)\;\rho \bar{v}\bar{Q},$$

where $\bar{v}=\frac{1}{2}\left(v_{1}+v_{2}\right)$. Finally,

(10) $$F_{d}=\frac{1}{2}\left(n_{2}-n_{1}\right)\;\rho \frac{\left(v_{1}+v_{2}\right)}{2}\left(A_{1}v_{1}+A_{2}v_{2}\right).$$

Force associated with tapering is

(11) $$F_{t}=F_{t}t,\;F_{t}=\int_{A_{2}}^{A_{1}}pdA,$$

where *p* is pressure and is written as $p=\frac{\rho {\bar{Q}}^{2}}{2}\left(\frac{1}{A_{2}^{2}}-\frac{1}{A^{2}}\right)$. Force due to taper is formulated as follows:

(12) $$F_{t}=\frac{\rho {\bar{Q}}^{2}}{2}\int_{A_{2}}^{A_{1}}\left(\frac{1}{A_{2}^{2}}-\frac{1}{A^{2}}\right)dA,$$

(13) $$F_{t}=\rho {\bar{Q}}^{2}\frac{{\left(A_{1}-A_{2}\right)}^{2}}{2A_{1}A_{2}^{2}}.$$

Total force acting on the element can now be written as follows:

(14) $$F_{e}=F_{f}+F_{d}+F_{t},$$

(15) $$F_{f}=t\;\pi \gamma \mu \left(u_{1}+u_{2}\right)h,$$

(16) $$F_{d}=\frac{1}{2}\left(n_{2}-n_{1}\right)\rho \left(v_{1}+v_{2}\right)\bar{Q},$$

(17) $$F_{t}=t\rho {\bar{Q}}^{2}\frac{{\left(A_{1}-A_{2}\right)}^{2}}{2A_{1}A_{2}^{2}}.$$

This force, calculated in each element of the vessel, contributes to the total force being applied on the head due to blood flow.

#### 3.2.1 Spatial mapping of elements

**Figure 8:**
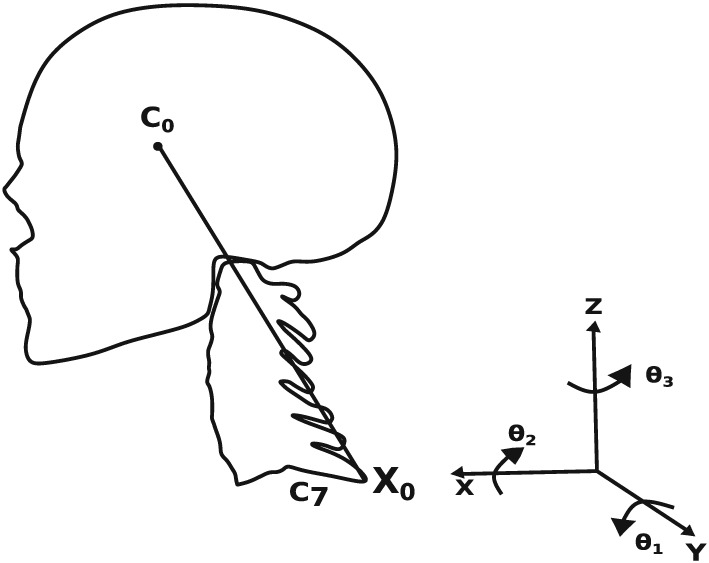
The head‐neck system employed in the present work

In order to link the blood flow and vibration models, all the individual finite elements in the carotid artery are identified with respect to the origin *C*7 in Figure 8. This is essential in order to calculate the moments about the origin for the vibration model, around which the head pivots. A CAD model developed from a scan was used to locate the relative locations of the finite elements along the carotid artery with respect to the pivot point.^33^

The morphology of carotid arteries varies among the general population, making patient‐specific spatial mapping of elements an impossible task without a CT‐scan of the head. By incorporating scans, the primary goal of developing a low cost and fast detection system goes unaccomplished. In order to solve this issue, the CAD model chosen was such that the morphology of carotids, by their relatively closer distance to the base of neck and smaller angle with respect to the reference axes (see Figure 8), produces the least amplitude possible for different stenoses conditions. By producing the least possible amplitudes, false negatives (subjects suffering from stenoses but detected to be healthy) can be minimised, as lower threshold values are chosen for categorising severity.

### 3.3 Dynamic equation of head‐neck system

A mathematical formulation is required to calculate the head‐neck motion. The dynamic equation formulated by Wang and Rahmatalla^34^ is used to analyse the head‐neck vibration induced by forces created from blood flow. The equation consists of the I_s_ matrix representing moments of inertia of the head and the first seven vertebral discs about the three primary axes (see Figure 8). Moments are applied by blood flow in the carotid arteries about X_0_ at C7. An assumption was made that the subject rests their back on a backrest allowing only for the head‐neck vibration to take place. This assumption helps to simplify the modelling, which would otherwise need a complex estimation of forces imparted in body parts below C7. For example, the force due to blood flow in major arteries such as the abdominal aorta, pumping of heart, and breathing. The dynamic equation used in the present study is as follows:

(18) $$I_{s}\ddot{\theta }+C_{s}\dot{\theta }+K_{s}\theta =M_{b},$$

where the inertia and stiffness matrices, I_s_ and K_s_ are defined as follows^34^:

$$I_{s}=\left[\begin{array}{ccc} LL_{c}mcos^{2}\theta_{20}+I_{11} & 0 & -\frac{1}{2}L_{c}mLcos\theta_{10}sin(2\theta_{20})\\ 0 & L_{c}mL+I_{22} & L_{c}mLsin\theta_{10}\\ -\frac{1}{2}L_{c}mLcos\theta_{10}sin(2\theta_{20}) & L_{c}mLsin\theta_{10} & b_{33} \end{array}\right],$$

where b_33_ is

$$b_{33}=\frac{3LL_{c}m}{4}-\frac{1}{4}mcos\left(2\theta_{10}\right)LL_{c}-\frac{1}{8}mcos\left(2\left(\theta_{10}-\theta_{20}\right)\right)LL_{c}-\frac{1}{4}mcos\left(2\theta_{10}\right)LL_{c}-\frac{1}{8}mcos\left(2\left(\theta_{10}+\theta_{20}\right)\right)L_{c}L+I_{33}$$

and

$$K_{s}=\left[\begin{array}{ccc} \frac{1}{2}\left(2K_{11}-2gL_{c}mcos\theta_{10}cos\theta_{20}\right) & \frac{1}{2}\left(2K_{12}+2gL_{c}msin\theta_{10}sin\theta_{20}\right) & K_{13}\\ \frac{1}{2}\left(2K_{12}+2gL_{c}msin\theta_{10}sin\theta_{20}\right) & \frac{1}{2}\left(2K_{22}-2gL_{c}mcos\theta_{10}cos\theta_{20}\right) & K_{23}\\ K_{13} & K_{23} & K_{33} \end{array}\right].$$

L_h_ and L_n_ are length of the head and neck respectively, L is the distance between C_0_ and C_7_, L_c_  =  m_n_ × L_n_ + m_h_ × L/m, and m is the sum of m_h_ and m_n_, masses of head and neck respectively. In the present work, m_h_ is kept constant (4.6 kg) but m_n_ is varied with body mass index (BMI). A linear change in mass of fat in the neck was adopted based on the change in neck circumference with BMI.^35^

The experimentally derived values for K_s_ and C_s_ (shown in Table 1) were adopted from Wang and Rahmatalla.^34^ Values for I_ii_, where i  =  1,2,3, were calculated from Himmetoglu et al.^36^ The subject's measured neck length (L_n_) and mass of the neck (m_n_) varied from 10 to 14 cm and 1.2 to 2 kg, respectively.The θ values depended on the orientation of the subject's head.

**Table 1:** Values for K_s_ and C_s_ from Wang and Rahmatalla^34^

Stiffness	k_11_	k_12_	k_13_	k_22_	k_23_	k_33_
(Nm/rad)	10.121	2.86	1.609	8.238	3.989	6.9867

Damper	c_11_	c_12_	c_13_	c_22_	c_23_	c_33_
(Nms/rad)	0.096	0.304	0.127	0.778	0.3034	−0.0689

### 3.4 Coupling moments calculated from haemodynamic model with the dynamic equation

Forces in each element are calculated from the haemodynamic model and are resolved into components parallel to the three axes.The forces in the cerebral arteries and other inter cranial arteries were accounted in our work by scaling the forces in last four elements situated in the end leading to the cerebral arteries.The force in these elements was scaled by seven times their original force. These elements were chosen to reflect an approximate distance of the cerebral arteries, the next major force contributing arteries after the carotid arteries, from X_0_. They are then used to find out moments, M_1_, M_2_ and M_3_ at X_0_ about the three principal axes. The calculated values form the moments matrix M_b_  =  [M_1_,M_2_,M_3_]^T^ are substituted into the dynamic equation, Equation 18. The completed dynamic equation is then solved for angular displacement using MATLAB's inbuilt ode solvers. The calculated angular displacements were further converted into linear displacements using the fixed distance between the forehead and X_0_, which were then projected onto a two‐dimensional plane using trigonometric relations, Y − Z (face). This projection was necessary to predict head‐neck motion along two axes as a single lens camera can capture only two dimensional arrays, leading to the tracking of points along two directions. An overview of the full algorithm from the video capture and modelling components, to the prediction is given in Figure 4.

### 3.5 Modelling stenoses

Deweese et al^37^ observed that the plaque build up starts about 1 cm before the bifurcation in the common carotid artery and extends up to 1.5 cm into the internal carotid artery. The haemodynamic model is modified to artificially represent the occlusion by altering the vessel geometry. In the one‐directional haemodynamic model, the diameter in the last centimetre in the common carotid arteries and the first one and a half centimetres of the internal and external carotid arteries was changed to values calculated from the following equation that depends on the percentage of blockage being analysed. The blockage in the left and right set of carotid arteries is set independently to accommodate different percentage of blockage in the respective arteries.

(19) $$\%\;Blockage=\frac{(internal\;diameter\;of\;vessel-diameter\;of\;lumen\;at\;maximum\;occlusion\;point)\times 100}{internal\;diameter\;of\;the\;vessel}.$$

Treatment of a stenosis in this manner assumes the most severe case possible, with the stenosis being treated a step decrease in the vessel area, which leads to the highest resistance estimation possible for a stenosis of a specific % blockage. The haemodynamic model described by Equations 1, 2, and 3 has previously been compared with three‐dimensional blood flow models for fractional flow reserve, in which the pressure drop across a coronary artery stenosis is estimated. Excellent agreement was observed between these one‐dimensional and three‐dimensional modelling methodologies.^4^, ^5^ During the simulation of the one‐dimensional blood flow model, the axial force is calculated as described in Section 3.2 which is coupled to the dynamic equation of the head‐next system, which is described in Section 3.3.

## 4 RESULTS AND DISCUSSIONS

The results presented here are the first attempt to validate the proposed model. Although a precise match of head vibration against synthetic data is unlikely, the clinically relevant data required may simply be the amplitude of the head vibration. If the amplitude of a head vibration can be linked directly to the severity of carotid stenosis, the impact on the patient treatment pathway could be hugely significant. For example, the technique could be used to prioritise patient treatment when screening patients who are at risk of stroke. Considering the potential of such a technique to separate healthy and unhealthy individuals, we have provided two categories of results in this work. In the first category, the proposed procedure is applied to a group of healthy volunteers, while the second category involves a patient with a severe carotid stenosis.

The healthy subjects are chosen with the assumption that volunteers aged between 20 and 30 years do not suffer from carotid stenoses. We believe that this assumption is valid in most cases (95.5%).^38^ In addition to healthy volunteers, we also had access to the data of a single patient with a severe carotid stenosis, left untreated. This patient previously suffered from a stroke, as a result of the carotid stenosis, and in order to access the data, we obtained the required signed consent from the patient via the treating clinician. All the data used are anonymous, and no personal information is disclosed at any point. The results of the model implemented in this work was not used in any way to inform patient treatment.

For both healthy subjects and the patient, individual face videos, weight, age, and height were recorded with their consent. Once the basic data are collected, the computed systemic arterial circulation and vibration models of the individuals are scaled to closely resemble reality. The synthetic data are then generated for normal and stenosed carotid arteries. When dealing with stenosed arteries, the severity of the stenosis is varied between 50% and 92%. The synthetic data thus generated are presented in the form of time‐dependent head vibrations. The synthetic vibrations are then compared against the measured vibrations to determine the approximate severity of the stenosis.

**Figure 9:**
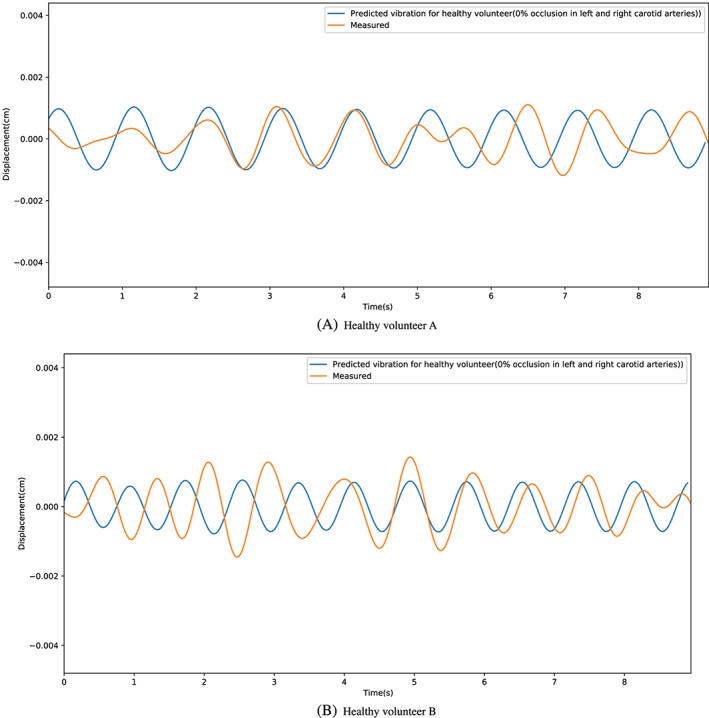
Comparison of synthetic and measured head vibration for healthy volunteers

**Figure 10:**
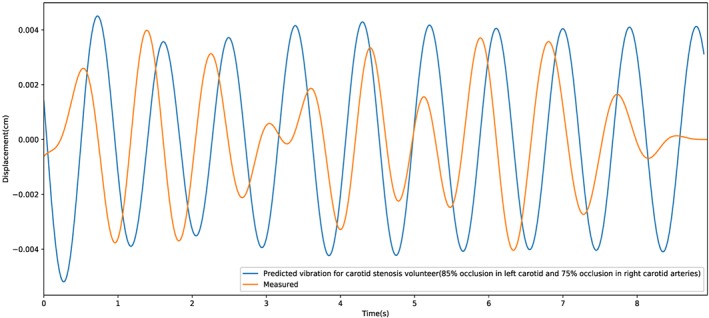
Comparison of synthetic and measured head vibration for a patient suffering from severe carotid occlusions

Figure 9 shows a comparison of head vibrations between synthetic and measured data. Excellent agreement is observed for both the magnitude and frequency of head vibrations. This indicates that the proposed method is correctly predicting the anticipated oscillations of the individual. Although not precise, an indicative prediction of severity is the starting point in noninvasively determining carotid occlusion. To further confirm the applicability of the proposed methodology, a video recording of a patient with a known carotid artery occlusion was taken. The comparison of in vivo measurements and synthetic data is shown in Figure 10. All occlusions below 50% may be classified as minor. Any blockage above this value is considered detectable using the proposed method. However, the signal from blockages above 70% may be harder to distinguish due to other cardiovascular issues such as cardiomyopathy. Thus, we categorise any occlusion above 70% as “requires attention.” In addition to assuming occlusions in only one of the carotid arteries, we require synthetic data that represent different combinations of occlusions in both left and right carotid arteries. Such data were created before the patient case mentioned was studied. The results as shown in Figure 10 matches excellently for frequency and magnitude. This patient has nearly 70% blockage in both left and right carotid arteries and thus was classified in the category of patients that require immediate attention.

### 4.1 Sensitivity analysis

**Figure 11:**
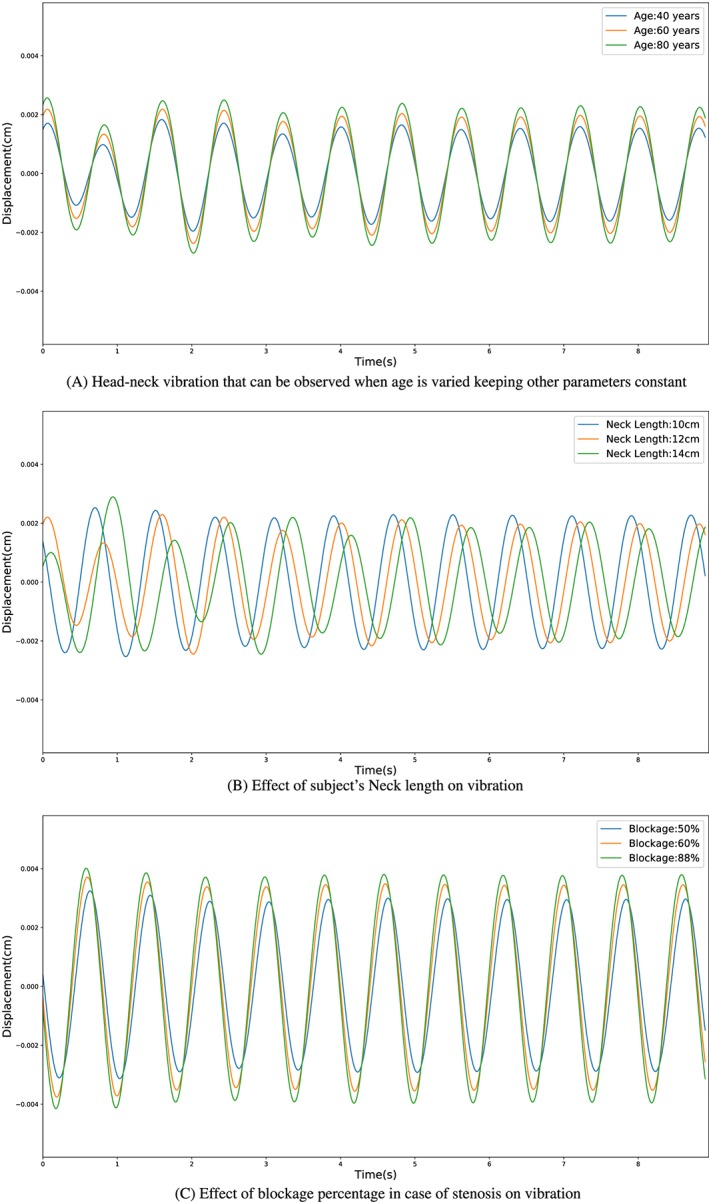
Sensitivity analysis of different input parameters on head‐neck vibration when other quantities have been kept constant

An analysis of the effect of each main input parameter on head‐neck vibration is necessary in order to understand their interactions within the model. Among the main parameters, three that need to be analysed critically are the input of age, neck length, and percentage of blockage. Age, which primarily affects the compliance of arteries, tends to cause a directly proportional increase in amplitude as the pulse wave velocity increases leading to increase in the friction force. Neck length, accounted in the dynamic equation, affects the amplitude by a significant amount. Variation of the blockage percentage is required to show how the mechanical model reacts to different severities of the stenosis. Figure 11 displays the effect of age, height, percentage of blockage (in the case of stenosis) when varied independently. For all simulations, the heart rate is kept consistently at 72 beats per minute, allowing a straightforward comparison of the cases considered.

The input for the age sensitivity tests is shown in Table 2. For the age‐related simulations, it is assumed that all individuals are healthy and do not have a stenosis. The pulse wave velocity estimation in the haemodynamic model increases with age. The amplitude of head oscillations predicted by the model in the virtual patient aged 80 years old are larger in magnitude than the 60‐year‐old, which in turn are larger in magnitude than the 40‐year‐old. This occurs even with a decrease in cardiac output for older individuals.

**Table 2:** Parameters of the sensitivity analysis for different ages

Age	Cardiac Output (*L*/*min*)	PWV (*m*/*s*)	MAP (*mmHg*)	HR (*bpm*)
40	7.57	7.09	95.5	72
60	7.25	8.88	92.9	72
80	6.98	10.72	90.6	72

Abbreviations: HR, heart rate; MAP, mean arterial pressure; PWV, pulse wave velocity.

The neck length varies the model predicted amplitude of vibration for the head‐neck system, and a phase shift is observed in Figure 11B. This indicates that an accurate measure of the neck length could be crucial for the dynamic model to accurately predict the magnitude of head‐neck oscillations. As expected, the amplitude of vibration increases with an increased percentage blockage. The amplitude increase from 0.0034 cm in the case of a 50% blockage, to 0.0041 cm in the case of an 80*%* blockage. Noting the significant increase in estimation from approximately a 0.002 cm (or less) amplitude of the head‐neck vibration in the healthy case, to over 0.003 for a 50*%* blockage, the model shows significant promise in potentially estimating the severity of a blockage in the carotid artery.

### 4.2 Discussions

The preliminary results presented in the previous section require further refinement. Although promising, the insufficient number of patients do not allow us to produce a precise categorisation at this stage. Another important observation made was that only blockages above 60% gave significantly higher vibration amplitudes than unblocked arteries. This rise in vibration amplitude is due to the increase in force imparted by the fluid on the plaque.

Another point that requires refinement is the location of the occlusions. The synthetic data generated assume that the locations of stenoses (both left and right side) have been fixed. By varying the locations of the stenoses, a large number of synthetic data can be generated but a manual comparison will then no longer be plausible. Thus, more advanced methods such as well‐trained machine learning methods to find the right match between synthetic and measured data would be required.

Another difficulty that arises in this work is the selection of components from the PCA for models with stenosis. The selection of the correct component for analysis is an absolute necessity as the magnitude of signal is important here for estimation of the blockage percentage. The most efficient way this selection can be performed is by retraining a machine learning algorithm using cardiac output, arterial condition, and physiological values from various patients as parameters. This will be possible only with the help of a large number of patient data, which will help us to produce a more accurate digital patient twin model.

## 5 LIMITATIONS

In this exploratory study, data from only one patient suffering from a stenosis were available. Typically someone suffering from a stenosis would undergo treatment to rectify this, and thus, it is extremely difficult to get access to patients between the diagnostic and the treatment stages. This limits the number of classifications we can generate, given the small cohort size. To approximately circumvent this, we have created virtual patients with artificial stenosis to indicate what the proposed methodology works in terms of head vibrations. In the one‐dimensional blood flow equations, a stenosis is currently added in the common, internal, and external carotid arteries via a step decrease in vessel diameter. This is a simplistic representation of what are often complex geometries. In reality, the stenoses may have varying geometric profiles that need more complex force estimations. There is a potential use for a scaling factor, which may aid in improving the accuracy of estimating these complex forces, and possibly take into account different geometries of plaque build up. However, this scaling factor needs to be calculated retrospectively by screening of TIA patients and potentially estimated by using machine learning techniques. The CAD model implemented is the same for all subjects. This is necessary; otherwise, a scan for each patient would be required, which would defeat the purpose of this study, which is to investigate an inexpensive and fast technique to detect carotid stenosis. However, we have chosen the geometry in such a way as to minimise the number of false negative predictions. This is achieved by choosing a geometry which would produce the least amplitude possible for the head‐neck vibration for both healthy and stenosed subjects.

## 6 CONCLUSIONS

A preliminary and very first attempt has been made to demonstrate that a coupled computer vision and computational mechanics model may be employed in the noninvasive detection of severe carotid stenosis. The results clearly indicate that the method proposed is viable, but it has room for substantial improvements. Both the healthy subject cases and a patient case presented provide us with sufficient confidence that the proposed noninvasive procedure is simple and fairly effective. Further development is required in order for the method to move towards a clinically usable platform. We believe that there are many steps that require development to realise the clinical potential and use of the proposed method. For example, a deep learning‐based automatic detection system is required in order to eliminate the manual comparison between synthetic head vibration data from the model and the in vivo head vibration captured through a video. The idea proposed here has a potential to noninvasively capture a large number of other blood flow related diseases if more sophisticated setups, such as multiple camera, accelerometer‐camera, and thermal imaging cameras can be used. Like any other new methodologies, a substantial study using patient data is necessary to proceed from research to implementation. With further progress, the proposed procedure can move towards an active human digital twin, in which continuous monitoring of carotid stenosis/stroke potential may take place.

## References

[1] Nithiarasu P . Active and Passive Human Digital Twins Based on Reduced Cardiovascular Flow Models BioMedEng 18 Conference Proceedings. London, UK: Imperial College; 2018 pp. 26.

[2] Nithiarasu P . Active and Passive Human Digital Twins—Future Prospects, VAJRA Colloquium Series: IIT Madras: Chennai, India Aug 7, 2018.

[3] Boileau E , Nithiarasu P . One‐dimensional modelling of the coronary circulation. Application to noninvasive quantification of fractional flow reserve (FFR) In: Tarves JMRS, Jorge RMN, eds. Computational and Experimental Biomedical Sciences: Methods and Applications. Azores: Springer; 2013:137‐156.

[4] Boileau E , Pant S , Roobottom C , et al. Estimating the accuracy of a reduced‐order model for the calculation of fractional flow reserve (FFR). Int J Numer Methods Biomed Eng. 2018;34(1):e2908 10.1002/cnm.2908 28600860

[5] Carson J , Pant S , Roobottom C , et al. Non‐invasive coronary CT angiography‐derived fractional flow reserve: a benchmark study comparing the diagnostic performance of four different computational frameworks. Int J Numer Methods Biomed Eng. 2018. 10.1002/cnm.3235 PMC6851543 31315158

[6] Mackay J , Mensah G , Mendis S , Greenlund K . The Atlas of Heart Disease and Stroke. Geneva: WHO; 2004.

[7] Stroke association . State of the Nation: Stroke Statistics (2018). London: Information guide, Stroke Association; Accessed January 26, 2018 https://www.stroke.org.uk/system/files/sotn_2018.pdf

[8] Shah S , Bellows BA , Adedipe AA , Totten JE , Backlund BH , Sajed D . Perceived barriers in the use of ultrasound in developing countries. Crit Ultrasound J. 2015;7(1):11. 10.1186/s13089-015-0028-2 PMC4485671 26123609

[9] Klingelhöfer J . Ultrasonography of carotid stenosis. IJCNMH. 2014;1(Suppl. 1):S04.

[10] Giovangrandi L , Inan OT , Wiard RM , Etemadi M , Kovacs GTA . Ballistocardiography—a method worth revisiting. In: 2011 Annual International Conference of the IEEE Engineering in Medicine and Biology Society, EMBC. Boston, MA, USA; 2011:4279‐4282. 10.1109/iembs.2011.6091062 PMC4274997 22255285

[11] He DD , Winokur ES , Sodini CG . A continuous, wearable, and wireless heart monitor using head ballistocardiogram (BCG) and head electrocardiogram (ECG). In: 2011 Annual International Conference of the IEEE engineering in medicine and biology society, Boston, MA, USA; 2011:4729‐4732. 10.1109/IEMBS.2011.6091171 22255394

[12] Balakrishnan G , Durand F , Guttag J . Detecting pulse from head motions in video 2013 IEEE Conference on Computer Vision and Pattern Recognition. 2013:3430‐3437.

[13] Bradski G . The OpenCV Library. Dr. Dobb's J Softw Tools. 2000;120:122‐125.

[14] Viola P , Jones M . Rapid object detection using a boosted cascade of simple features. In: Proceedings of the 2001 IEEE Computer Society Conference on Computer Vision and Pattern Recognition. CVPR 2001, Vol. 1. Kauai, HI, USA; 2001:I-I. 10.1109/CVPR.2001.990517

[15] Haque MA , Nasrollahi K , Moeslund TB , Irani R . Heartbeat rate measurement from facial video. IEEE Intell Syst. 2016;31:40‐48.

[16] Li X , Chen J , Zhao G , Pietikäinen M . Remote heart rate measurement from face videos under realistic situations. In: IEEE Conference on Computer Vision and Pattern Recognition Columbus, OH: Columbus, OH; 2014:4264‐4271.

[17] Irani R , Nasrollahi K , Moeslund TB . Improved pulse detection from head motions using DCT. In: 2014 International Conference on Computer Vision Theory and Applications (VISAPP), Vol. 3. Lisbon, Portugal; 2014:118‐124.

[18] Shan L , Yu M . Video‐based heart rate measurement using head motion tracking and ICA. In: IEEE 6th International Congress on Image and Signal Processing (CISP), Vol. 1. Hangzhou, China; 2013:160‐164.

[19] Mynard JP , Smolich JJ . One‐dimensional haemodynamic modeling and wave dynamics in the entire adult circulation. Ann Biomed Eng. 2015;43(6):1443‐60. 10.1007/s10439-015-1313-8 25832485

[20] Mynard JP , Nithiarasu P . A 1D arterial blood flow model incorporating ventricular pressure, aortic valve and regional coronary flow using locally conservative Galerkin (LCG) method. Commun Numer Methods Eng. 2008;24:367‐417.

[21] Low K , van Loon R , Sazonov I , Bevan RLT , Nithiarasu P . An improved baseline model for a human arterial network to study the impact of aneurysms on pressure‐flow waveforms. Int J Numer Methods Biomed Eng. 2012;28:1224‐1246. 10.1002/cnm.2533 23212798

[22] Müller LO , Toro FE . Well‐balanced high‐order solver for blood flow in networks of vessels with variable properties. Int J Numer Methods Biomed Eng. 2013;29:1388‐1411. 10.1002/cnm.2580 23913466

[23] Müller LO , Toro EF . A global multiscale mathematical model for the human circulation with emphasis on the venous system. Int J Numer Methods Biomed Eng. 2014;30:681‐725. 10.1002/cnm.2622 24431098

[24] Alastruey J , Hunt AAE , Weinberg PD . Novel wave intensity analysis of arterial pulse wave propagation accounting for peripheral reflections. Int J Numer Methods Biomed Eng. 2014;30:249‐279. 10.1002/cnm.2602 PMC4297358 24132888

[25] Keijsers JMT , Leguy CAD , Huberts W , Narracott AJ , Rittweger J , van de Vosse FN . A 1d pulse wave propagation model of the hemodynamics of calf muscle pump function. Int J Numer Methods Biomed Eng. 2015;31:e02714 10.1002/cnm.2714 PMC4676919 25766693

[26] Huang PG , Müller LO . Simulation of one‐dimensional blood flow in networks of human vessels using a novel TVD scheme. Int J Numer Methods Biomed Eng. 2015;31:e02701 10.1002/cnm.2701 25529823

[27] Trenhago PR , Fernandes LG , Muller LO , Blanco PJ , Feijo RA . An integrated mathematical model of the cardiovascular and respiratory systems. Int J Numer Methods Biomed Eng. 2015;32:e02736 10.1002/cnm.2736 26198626

[28] Boileau E , Nithiarasu P , Blanco JB , et al. A benchmark study of one‐dimensional numerical schemes for arterial blood flow modelling. Int J Numer Methods Biomed Eng. 2015;31:e02732 10.1002/cnm.2732 26100764

[29] Carson J , Van Loon R . An implicit solver for 1D arterial network models. Int J Numer Methods Biomed Eng. 2017;33:e2837. 10.1002/cnm.2837 27709800

[30] Müller LO , Blanco PJ , Watanabe SM , Feijóo RA . A high‐order local time stepping finite volume solver for one‐dimensional blood flow simulations: application to the adan model. Int J Numer Methods Biomed Eng. 2016;32:e02761 10.1002/cnm.2761 26695621

[31] Danilov A , Ivanov Y , Pryamonosov R , Vassilevski Y . Methods of graph network reconstruction in personalized medicine. Int J Numer Methods Biomed Eng. 2016;32:e02754 10.1002/cnm.2754 26462139

[32] Passera K , Manini S , Antiga L , Remuzzi A . Patient‐specific model of arterial circulation for surgical planning of vascular access. J Vasc Access. 2013;14(2):180‐192. 23032951 10.5301/jva.5000099

[33] Org PrintHuman . Partial Brain Vascular Network,https://3dprint.nih.gov/discover/3dpx-001277; Accessed on: Jan 2018.

[34] Wang Y , Rahmatalla S . Human head‐neck models in whole‐body vibration: effect of posture. J Biomech. 2013;46:702‐710. 23290314 10.1016/j.jbiomech.2012.11.037

[35] Hingorjo MR , Quereshi MA , Mehdi A . Neck circumference as a useful marker of obesity: a comparison with body mass index and waist circumference. J Pak Med Assoc. 2012;62(1):36‐40. 22352099

[36] Himmetoglu S , Acar M , Taylor AJ , Bouazza‐Marouf K . A multi‐body head‐and‐neck model for simulation of rear impact in cars. Proc Inst Mech Eng D‐J Auto Eng. 2007;221:15.

[37] Deweese JA , May AG , Lipchik EO , Rob CG . Anatomic and hemodynamic correlations in carotid artery stenosis. Stroke. 1970;1(3):149‐157. 5522912 10.1161/01.str.1.3.149

[38] Sturlaugsdottir R , Aspelund T , Bjornsdottir G , et al. Prevalence and determinants of carotid plaque in the cross‐sectional refine‐reykjavik study. BMJ Open. 2016;6:e012457 10.1136/bmjopen-2016-012457 PMC5168519 27884845

